# Protein cross-linking by chlorinated polyamines and transglutamylation stabilizes neutrophil extracellular traps

**DOI:** 10.1038/cddis.2016.200

**Published:** 2016-08-11

**Authors:** Krisztián Csomós, Endre Kristóf, Bernadett Jakob, István Csomós, György Kovács, Omri Rotem, Judit Hodrea, Zsuzsa Bagoly, Laszlo Muszbek, Zoltán Balajthy, Éva Csősz, László Fésüs

**Affiliations:** 1 Department of Biochemistry and Molecular Biology, University of Debrecen, Debrecen, Hungary; 2 Department of Biophysics and Cell Biology, University of Debrecen, Debrecen, Hungary; 3 Department of Computer Graphics and Image Processing, University of Debrecen, Debrecen, Hungary; 4 Division of Clinical Laboratory Science, University of Debrecen, Debrecen, Hungary; 5 Thrombosis and Hemostasis Research Group of the Hungarian Academy of Sciences, University of Debrecen, Debrecen, Hungary

## Abstract

Neutrophil extracellular trap (NET) ejected from activated dying neutrophils is a highly ordered structure of DNA and selected proteins capable to eliminate pathogenic microorganisms. Biochemical determinants of the non-randomly formed stable NETs have not been revealed so far. Studying the formation of human NETs we have observed that polyamines were incorporated into the NET. Inhibition of myeloperoxidase, which is essential for NET formation and can generate reactive chlorinated polyamines through hypochlorous acid, decreased polyamine incorporation. Addition of exogenous primary amines that similarly to polyamines inhibit reactions catalyzed by the protein cross-linker transglutaminases (TGases) has similar effect. Proteomic analysis of the highly reproducible pattern of NET components revealed cross-linking of NET proteins through chlorinated polyamines and *ɛ*(*γ*-glutamyl)lysine as well as bis-*γ*-glutamyl polyamine bonds catalyzed by the TGases detected in neutrophils. Competitive inhibition of protein cross-linking by monoamines disturbed the cross-linking pattern of NET proteins, which resulted in the loss of the ordered structure of the NET and significantly reduced capacity to trap bacteria. Our findings provide explanation of how NETs are formed in a reproducible and ordered manner to efficiently neutralize microorganisms at the first defense line of the innate immune system.

Neutrophils act as the first line of defense in the immune response against microbes and parasites. In addition to phagocytosis, generation of reactive oxygen species (ROS) and antimicrobial macromolecules, neutrophil extracellular trap (NET) was discovered more than a decade ago as an additional neutrophil task.^1^ Upon various inflammatory stimuli, neutrophils become activated and undergo a specific form of cell death (termed NETosis) in which they eject preprocessed DNA content in the form of a web-like structure thereby having the ability to trap, neutralize and eradicate invading pathogens. The molecular mechanism of NETosis involves stimulation of neutrophil surface receptors (Toll-like receptors (TLRs), Fc and/or complement) leading to elevation of intracellular calcium, activation of protein kinase C and formation of ROS.^2^ Subsequent downstream events lead to changes in the composition of the cytoplasm, in which disintegration of the nuclear and granular membranes allows mixing of their contents. Unwinding of the chromatin appears to be dependent on histone deamination catalyzed by peptidylarginine deiminase 4 (PAD4), eventually culminating in extrusion of a characteristic NET structure into the extracellular space.

The NET itself is a complex and highly organized extracellular structure composed of a DNA backbone of interwoven filaments with a diameter of 15–17 nm containing stacked nucleosomes.^3^ The strands aggregate into larger threads with a diameter of 50 nm and can further form much larger three-dimensional web-like structures, measuring hundreds of nanometers in length and width. In addition to the function of the NET as a physical barrier, the DNA backbone is embedded with a number of peptides, proteases (neutrophil elastase (NE), myeloperoxidase (MPO) and bactericidal permeability increasing protein) and histones (H1, H2A, H2B, H3 and H4) contributing to the direct microbicidal effect of the NET. Molecular determinants of the reproducibly formed, well-ordered NET architecture have not been revealed yet.

Examination of the three-dimensional structure, the non-random orientation of its fibers and the overall stability of the NET has directed our attention to specific covalent protein–protein cross-link formation, which may determine these features. Primary amines (including polyamines) were found to be chemically incorporated into proteins through mono- and dichloramine intermediates generated in the presence of hypochlorous acid (HOCl).^4^ Neutrophil NADPH oxidase and MPO generate a highly oxidative state in activated neutrophils, which is necessary for incorporation of chlorinated amines.^5, 6^ Indeed, more recently, intracellular MPO activity was implicated in functional NET formation at a cell-autonomous but so far uncharacterized way.^7^ Covalent bonds can be generated enzymatically by transglutaminases (TGases) that are Ca^2^-dependent enzymes and able to form protease resistant *ε*-(*γ*-glutamyl)lysine cross-links between the *γ*-carboxamido group of glutamine residues and the *ε*-amino group of lysine residues in polypeptides. Members of the TGase family have been implicated in the organization and stabilization of highly organized protein assembles, such as the fibrin clot, the extracellular matrix and the cornified envelope in the skin.^8, 9, 10, 11, 12, 13, 14, 15, 16, 17, 18, 19, 20, 21^ In the presence of naturally occurring di- and polyamines (putrescine (PUT), spermidine (SPD) and spermine (SPM)), TGases also catalyze amine incorporation into spatially accessible glutamines in substrate proteins resulting in mono(*γ*-glutamyl)- or bis(*γ*-glutamyl)-PUT, -SPD or-SPM.^22, 23, 24^ Here we show that cross-link formation between NET-specific proteins by chlorinated polyamines and TGase-catalyzed reaction in activated neutrophils are necessary to form functional NETs.

## Results

### Covalent incorporation of endogenous polyamines into neutrophil proteins during NETosis

**Figure 1:**
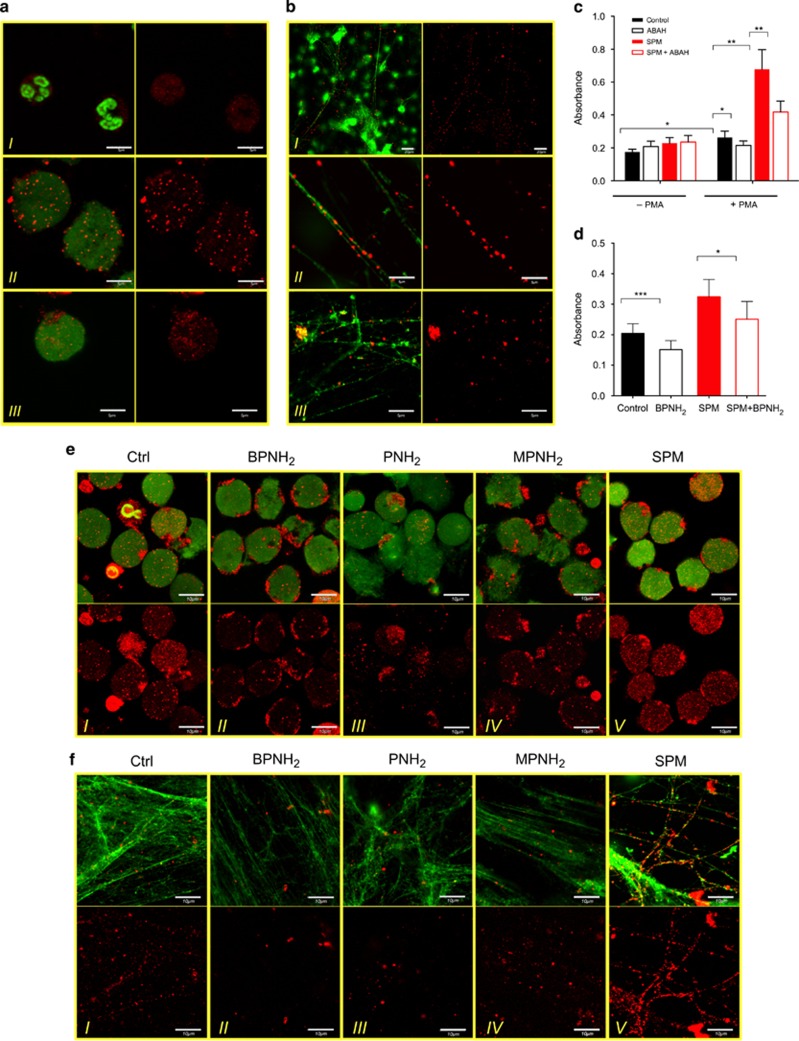
Endogenous and exogenous polyamines are incorporated into cellular and NET-specific proteins during NET formation. (**a**) Representative images of SPD and SPM distribution in resting (I), 4 h PMA-(II) and *S. aureus* (III) activated human neutrophils. Left panels show polyamines (red) and DNA (green); right panels show polyamine staining alone. Experiment was repeated at least six times with neutrophils from independent healthy donors, with similar results. Original magnification was × 60, scale bar represents 5 *μ*m. (**b**) Representative images of SPD and SPM content of NET. Neutrophils were treated with PMA (I–II) or *S. aureus* (III) and stained as in **a**. Left panels show polyamines (red) and DNA (green); right panel shows polyamines alone. Experiment was repeated at least six times with neutrophils from independent healthy donors, with similar results. Original magnifications were × 40 (I) or × 60 (II and III) and scale bars represent 20 *μ*m (I) or 5 *μ*m (II and III). (**c**) Detection of polyamine incorporation into cellular proteins with ELISA. Resting and PMA-activated neutrophils were cultured alone or in the presence of SPM, ABAH (MPO inhibitor) or SPM and ABAH and then covalently conjugated polyamines were quantified from cell lysate. (**d**) Detection of polyamine incorporation into NET-specific proteins with ELISA. NET formation was induced alone or in the presence of SPM, BPNH_2_ or SPM and BPNH_2_ and then covalently conjugated polyamines to proteins were quantified. ELISA experiments for cellular and NET proteins were performed on neutrophils isolated from five independent healthy donors in duplicates. Data are presented as mean±S.E.M. Differences on treatments were tested for significance with paired Student's *t*-test. *P<0.05; **P<0.01; ***P<0.001. (**e**) Representative images of SPD/SPM distribution in neutrophils activated for 4 h with PMA alone (I), or in the presence of BPNH_2_ (II), PNH_2_ (III), MPNH_2_ (IV) or SPM (V). Upper panels show polyamines (red) and DNA (green); lower panels show polyamine staining alone. (**f**) Representative images of SPD/SPM content of the NET from neutrophils treated and stained as in Figure 2a. Upper panels show polyamines (red) and DNA (green); lower panels show polyamine staining alone. Experiment was repeated at least four times with neutrophils from independent healthy donors, with similar results. Original magnification was × 60, scale bar represents 10 *μ*m

In order to investigate whether endogenous polyamines may have a role in the formation of NET, we first attempted to detect SPM and SPD covalently bound to cellular proteins in resting and activated NET-forming human neutrophils by confocal microscopy. In resting neutrophils, covalently bound SPM and SPD were hardly detectable. In contrast, activation of neutrophils either with the protein kinase C activator, phorbol-12-myristate 13-acetate (PMA), or with the live pathogen *Staphylococcus aureus* revealed a strong dotted pattern in polyamine staining. The size of SPM and SPD-rich speckles was in the range of 1–2 *μ*m and the dots were localized in the area occupied by the decondensed chromatin (Figure 1a). Interestingly, polyamine speckles along with the NET DNA filaments were also detected suggesting that endogenous polyamines get incorporated into the protein fraction of the NET too (Figure 1b).

Using ELISA, we detected an increase in the level of covalently bound polyamines (SPM and SPD) to cellular protein of PMA-stimulated neutrophils. This increase could be reverted in the presence of aminobenzoic acid hydrazide (ABAH), a MPO inhibitor. Covalent incorporation of exogenously administered SPM was also observed occurring in a partially MPO-dependent manner as indicated by its ABAH inhibition (Figure 1c). Endogenous polyamines as well as added SPM could also be detected in proteins isolated from the NET (Figure 1d). As MPO function is essential for the entire NET formation, it is not possible to utilize ABAH to investigate the role of MPO in polyamine incorporation into NET proteins. Therefore, we tested the effect of the 5-(biotinamido)-pentylamine (BPNH_2_) as a potential competitive monoamine inhibitor of polyamine incorporation.^25^ BPNH_2_ efficiently reduced the level of both endogenous and extraneous SPM in NET proteins (Figure 1d).

Next, we tested polyamine conjugation to cellular and NET proteins in the presence of different monoamine derivatives with immunostaining. Besides BPNH_2_, we used pentylamine (PNH_2_) as an unmodified monoamine and *N*-methylpentylamine (MPNH_2_) as a negative control. As Figure 1e shows both monoamines with free terminal amino groups (BPNH_2_ and PNH_2_) interfered with endogenous polyamine incorporation into cellular proteins, as a reduced number of polyamine containing dots was detectable inside the NET-forming neutrophils (Figure 1e, panels I–III). MPNH_2_ that lacks a terminal amino group did not influence endogenous polyamine incorporation levels (Figure 1e, panel IV), although it had similar inhibitory effect on ROS generation as PNH_2_ (Supplementary Figure 1). Furthermore, although overloading the neutrophils with SPM also caused reduced ROS production (Supplementary Figure 1),^26, 27^ it resulted in a significant increase in the number of detectable covalently bound polyamine containing dots (Figure 1e, panel V). Polyamine content of NET-specific proteins was also reduced in the presence of monoamines with a reactive terminal amino group (BPNH_2_ and PNH_2_), whereas MPNH_2_ did not have any effect (Figure 1f, panels I–V). In addition, exogenous SPM was again notably incorporated into NET-specific proteins along the strands of the NET structure (Figure 1f, panel V).

These data indicate that upon NET formation endogenous polyamines are covalently incorporated into cellular and NET-specific proteins that process depends on MPO activity. Also, the level of the protein-bound polyamines can be increased by the administration of exogenous SPM and reduced by monoamine treatment.

### Monoamines mimic endogenous polyamine incorporation in NETosing neutrophils

**Figure 2:**
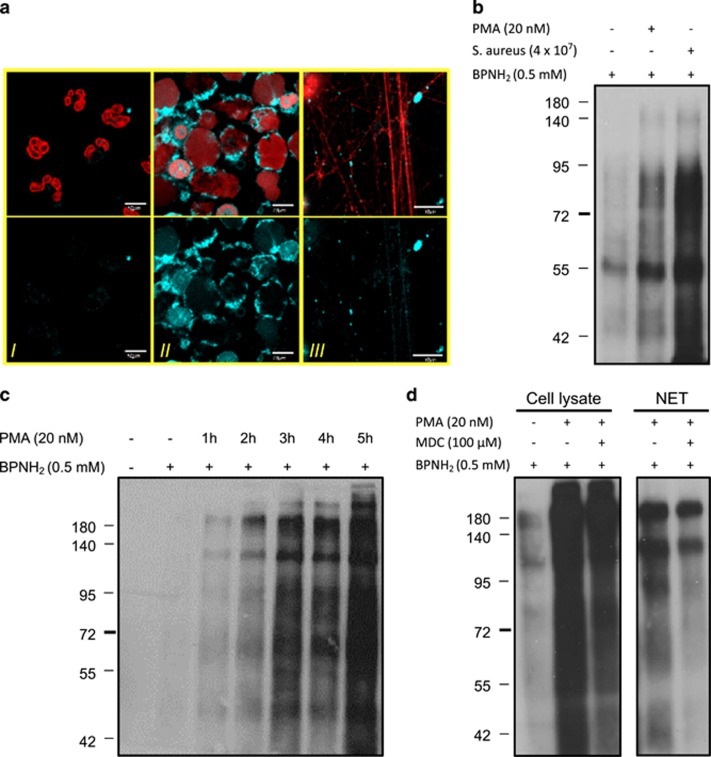
Exogenous monoamines mimic endogenous polyamine incorporation occurring in NETosing neutrophils. (**a**) Representative images of BPNH_2_ incorporation into neutrophil intracellular and NET proteins upon 4-h activation with PMA. Neutrophils from healthy donors were preincubated with BPNH_2_ for 1 h and then stimulated with PMA or left unstimulated for 4 h (control). (I) Shows BPNH_2_ incorporation in resting neutrophils; (II) shows BPNH_2_ incorporation in activated neutrophils; and (III) shows BPNH_2_ incorporation into NET proteins. Upper panels show BPNH_2_ (light blue) and DNA (red); lower panels show BPNH_2_ staining alone. The experiment was repeated at least three times with neutrophils from independent healthy donors, with similar results. The original magnification was × 60, the scale bar represents 10 *μ*m. (**b**) Detection of exogenous monoamine (BPNH_2_) incorporation into cellular proteins in activated neutrophils by western blot. Neutrophils from a healthy donor were preincubated with BPNH_2_ and then stimulated either with PMA or *S. aureus* or left unstimulated for 4 h (control). Levels of BPNH_2_ incorporation into cellular proteins were determined from cell lysate with western blot. (**c**) Time-dependent incorporation of BPNH_2_ into neutrophil cellular proteins upon activation. Neutrophils from a healthy donor were preincubated with BPNH_2_ and then stimulated with PMA for 0–5 h. BPNH_2_ incorporation levels were detected as in Figure 2b. (**d**) Effect of MDC on BPNH_2_ incorporation into cellular and NET proteins. Neutrophils were preincubated with BPNH_2_ or BPNH_2_ and MDC followed by PMA stimulation for 4 h or left unstimulated (control). Cellular and NET proteins were isolated and BPNH_2_ incorporation levels were detected as in Figure 2b

To characterize monoamine incorporation into cellular and NET-specific proteins, we induced NET formation in the presence of BPNH_2._ Proteins covalently labeled with BPNH_2_ were detected in PMA-activated neutrophils, whereas cellular proteins in resting cells remained unconjugated (Figure 2a, panels II and I, respectively). Proteins localized in the NET structure were also labeled with BPNH_2_ (Figure 2a, panel III). Based on the same pattern of monoamine incorporation (BPNH_2_) and of endogenous polyamines detected by immunohistochemistry, we assumed that monoamine incorporation can be used as a tool to further characterize amine incorporation.

As a next step, protein extracts from neutrophils were separated in sodium dodecyl sulfate (SDS)–polyacrylamide gels and proteins into which BPNH_2_ had been incorporated were visualized by western blot using HRP-conjugated streptavidin. As Figure 2b shows, upon PMA stimulation several cellular proteins labeled with BPNH_2_ were detectable at various molecular weights. A stronger modification was observed when neutrophils were activated with pathogen stimulation (Figure 2b). BPNH_2_ incorporation continuously increased in time during neutrophil activation (Figure 2c). Treatment with a polyamine derivative, monodansylcadaverine (MDC) resulted in a notable reduction in BPNH_2_ incorporation in cellular and as well as NET proteins suggesting competition between monoamines and polyamines for target proteins (Figure 2d).

### Proteomic analysis of the NET reveals polyamine-mediated protein cross-links by chlorinated polyamines and TGase reaction

**Figure 3:**
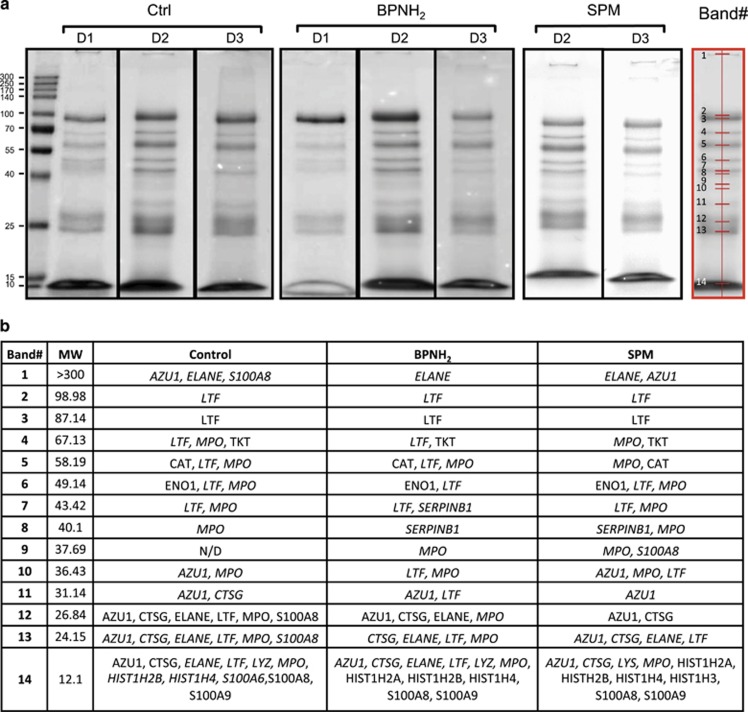
Proteomic analysis of the NET confirms protein cross-linking. (**a**) SDS-PAGE of untreated (Ctrl), BPNH_2_ and SPM treated NET proteins. In all, 20 *μ*g of protein from each sample was subjected to SDS-PAGE on a 12% acrilamide gel for 1.5 h at 30 mA constant current and stained with PageBlue Protein Staining Solution. The detected bands and the band numbers assigned by QuantiyOne (Bio-Rad) band analysis software are marked with red. D1, D2 and D3 indicate three independent healthy donors involved in the analysis. (**b**) List of proteins identified by LC-MS/MS based mass spectrometry analysis in each band. Italics indicate the presence of proteins at molecular weights different from the expected. The Uniprot gene name of identified proteins is presented in the table. The estimated MW values are also indicated. For protein identification details, see Supplementary Table 1

Next, we analyzed protein fraction of NET with SDS-polyacrilamide gel electrophoresis (PAGE) and identified a highly reproducible pattern in the protein composition of NET (Figure 3a). By mass spectrometry, we could identify 16 NET proteins 14 of which (azurocidin AZU1, NE, protein S100-A8, protein S100-A9, lactotrasferrin, MPO, transketolase, catalase, alpha-enolase, cathepsin G, lysozyme C, histone H2A, histone H2B, histone H3 and histone H4) have already been described,^3^ whereas 2 (leukocyte elastase inhibitor 1 and protein S100-A6) have not been reported as NET components so far. Gel image analysis of untreated, BPNH_2_ and SPM-treated NET proteins revealed that although the pattern of protein composition was not different, alterations in band intensities upon BPNH_2_ and SPM treatment occurred (Figure 3a). Subsequent LC-MS/MS based identification of NET proteins (Supplementary Table 1) showed that while some proteins were identified in bands corresponding to their theoretical molecular weights, many migrated to different positions (Figure 3b) indicating extensive protein processing.

**Figure 4:**
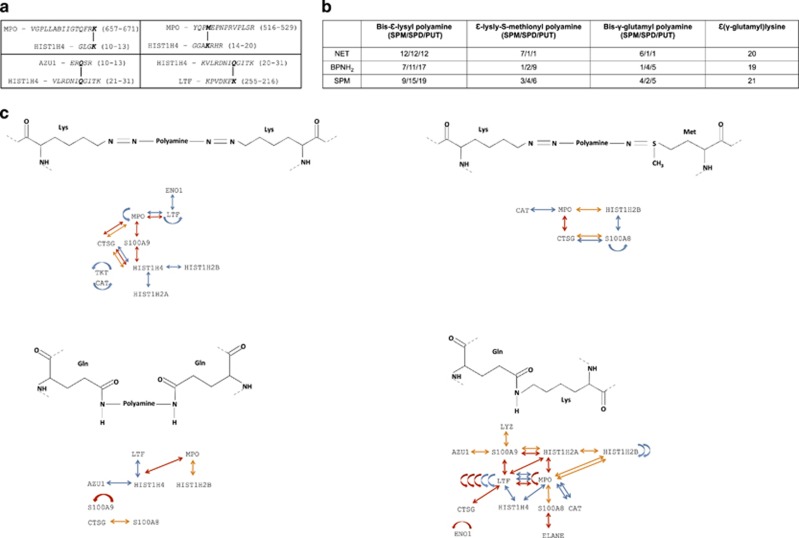
Analysis of protein cross-links between the identified NET proteins. (**a**) Selected examples for cross-links formed between the identified NET proteins. (**b**) The number of cross-links identified by the Stavrox software. The values apply to the number of cross-links confirmed by three independent Stavrox analyzes. (**c**) Representation of the network of cross-linked NET proteins obtained from donor 3. Blue arrows indicate NET protein cross-links within untreated samples; red arrows show cross-links among NET proteins upon BPNH_2_ treatment, whereas the orange arrows represent the protein cross-links formed upon SPM treatment. Curved arrows indicate cross-links formed between copies of the same protein. In case of the presented cross-linked proteins, the Uniprot gene names are indicated. For raw data, see Supplementary Table 2

The mass spectrometry data obtained from identified proteins were examined with the software, StavroX^28^ for the possible presence of bis-*ɛ*-lysyl polyamine and *ɛ*-lysyl-S-methionyl polyamine cross-links, which can be generated by chlorinated polyamines^4^ as well as bis-*γ*-glutamyl polyamine and *ɛ*(*γ*-glutamyl)lysine cross-links formed by TGase reaction^29, 30^ (Figures 4a and b). The four types of cross-links formed between specific residues of the NET proteins could be found (Figures 4a and b) as detailed in Supplementary data set (http://ngsdebftp.med.unideb.hu/proteomics/).

Mass spectrometry data were examined with the software, StavroX, to identify cross-links formed at specific side chains between the recognized proteins.^31^ We could find bis-*ɛ*-lysyl polyamine and *ɛ*-lysyl-S-methionyl polyamine cross-links formed by chlorinated polyamine incorporation and bis-*γ*-glutamyl polyamine as well as *ɛ*(*γ*-glutamyl)lysine cross-links typically catalyzed by a TGase type reaction (Figures 4a and b,Supplementary Table 1). The detected number of cross-links was similar or somewhat lower in BPNH_2_-treated samples (except for PUT derivatives, which were increased) and SPM-treated samples yielded an increased number of this type of cross-links. A similar tendency was observed regarding the number of TGase formed bis-*γ*-glutamyl polyamine and *ɛ*(*γ*-glutamyl)lysine cross-links (Figure 4b). Representation of the pattern of detected cross-links revealed a polyamine and *ɛ*(*γ*-glutamyl)lysine-mediated covalent network formed among NET proteins and also reveals changes in the position of cross-links upon BNPH_2_ and SPM treatment (Figure 4c,Supplementary Table 2) when different cross-links are formed at different positions resulting in alterations of the network of cross-linked NET proteins.

### Potential role of TGase 1 in cross-link formation in NETosis

**Figure 5:**
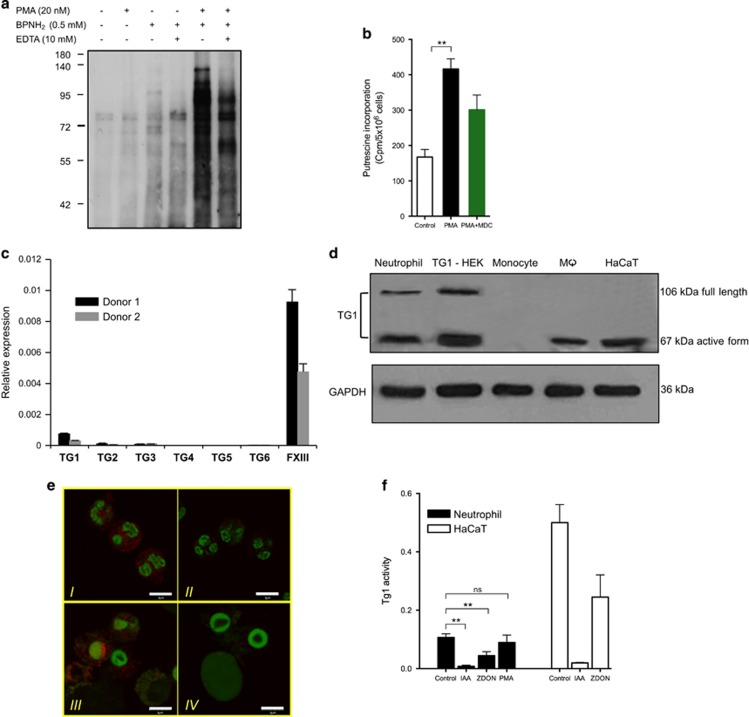
Detectable expression and activity of TG1 in NETosing neutrophils. (**a**) Calcium dependence of BPNH_2_ incorporation into cellular proteins. NETosis was initiated in neutrophils with PMA for 2 h in the presence of Ca^2+^, and then cells were either treated with EDTA for 30 min or left untreated. BPNH_2_ was given to the pretreated cells for additional 2 h and finally its incorporation levels were detected similar to the previous experiments. (**b**) Detection of exogenous polyamine (PUT) incorporation into cellular proteins in activated neutrophils by *in situ* radioactive filter paper TGase activity assay. Neutrophils from a healthy donors were preincubated with [3H]putrescine and then stimulated either with PMA or PMA and MDC or left unstimulated for 4 h (control). Level of [3H]putrescine incorporation into cellular proteins was determined from cell lysate. [3H]putrescine incorporation in PMA-activated neutrophils was significantly increased and slightly diminished in the presence of MDC. Data are represented as mean±S.E.M. based on two independent healthy donors with three parallels in each experiment. (Paired Student's *t*-test, **P<0.01). (**c**) Detection of TG1, TG2, TG3, TG4, TG5, TG6 and Factor XIII expression in neutrophils by real-time qPCR. Neutrophils were isolated from two independent healthy donors and relative mRNA levels of TGases were determined using cyclophillin A transcript as reference. (**d**) Detection of TG1 at the protein level in neutrophils. Neutrophils and other human myeloid cells were lysed and TG1 protein levels were detected by western blot. HaCaT and TG1 overexpressing HEK cells were used as positive controls. The same bands, corresponding to the full length and the protease cleaved short forms of TG1 (ref 29), could be observed in the neutrophil cell lysate as in the HEK cells overexpressing TG1. GAPDH level was detected as loading control. (**e**) Representative images of imunofluorescent staining of TG1 in human neutrophils. Resting (I and II) and PMA-activated (III and IV) neutrophils were stained with an anti-TG1 rabbit polyclonal antibody and Alexa Fluor-647-conjugated goat anti-rabbit secondary antibody (red) and SYTO 83 orange fluorescent nucleic acid stain to visualize DNA (green). Images II and VI show the negative control for TG1 staining, where the primary antibody was omitted. The experiment was repeated at least four times with neutrophils from independent healthy donors, with similar results. The original magnification was × 60, the scale bar represents 10 *μ*m. (**f**) Detection of specific TG1 activity in neutrophil cell lysate. TG1 activity was detected by ELISA in resting and PMA-activated neutrophils and HaCaT (positive control) cell lysates alone or in the presence of the non-specific irreversible TGase inhibitor IAA or the specific irreversible TG2 and FXIII-A inhibitor (ZDON, not tested previously for TG1 inhibition) using a TG1-specific substrate peptide

Based on the proteomic data, we propose that besides chlorinated polyamine-mediated mechanisms enzymatic polyamination and protein cross-linking by TGase also occur in activated neutrophils. Therefore, we tested if the amine incorporation depends on the presence of Ca^2+^ as a hallmark of TGase-mediated reaction. As NET formation in itself is Ca^2+^ dependent, first we initiated NETosis with PMA in the presence of Ca^2+^. After 2 h, calcium ion was scavenged from the culture medium with ethylenediaminetetraacetic acid (EDTA) for 30 min, then BPNH_2_ was given to NETosing cells for 3 h. As a result, significant decrease in the amount of monoamine-conjugated proteins was observed in samples lacking Ca^2+^ in the last 3 h of NETosis (Figure 5a, lines 4 and 6). To further investigate possible TGase catalyzed amine incorporation, we modified a radioactive filter paper-based TGase activity assay^32^ to demonstrate PUT conjugation as alternative exogenous polyamine incorporation. After inducing NETosis detection of conjugated [3H]putrescine to proteins in neutrophil lysates showed a significant increase (Figure 5b). Incorporation was diminished in the presence of MDC. These observations suggest enzymatically mediated conjugation of mono- and polyamines by a TGase supplementing the chlorination driven amine incorporation.

Next, in a search for the potential presence of TGases, the expression of TG1-6 and Factor XIII-A expression levels were measured. From the seven investigated variants, TG1 and Factor XIII-A were found to be transcribed at significant levels (Figure 5c), whereas the ubiquitous TG2 was not detectable at mRNA (Figure 5c) or at protein level (data not shown). Interestingly, we managed to detect TG1 by immunoblotting and immunohistochemistry (Figures 5d and e). TG1 must be activated by proteases;^31^ the cleaved active form was detected similarly as in TG1-transfected cells. Moreover, specific TG1 activity could be measured in neutrophil lysate utilizing a TG1-specific glutamine substrate (Figure 5f) indicating that it can potentially contribute to polyamine incorporation and protein cross-linking during NETosis.

To demonstrate FXIII-A expression, we performed immunohistochemistry analysis on neutrophils isolated from healthy individuals as well as a FXIII-A-deficient patient. In neutrophils from a healthy donor, FXIII-A could be visualized at a lower level than in monocytes, whereas in the neutrophils or monocytes of the FXIII-A-deficient patient, we failed to detect any specific staining in neutrophils or monocytes (Supplementary Figure 2A). Staining pattern of FXIII-A in neutrophils from healthy donors showed granular localization suggesting that FXIII-A content of neutrophils may derive from phagocytosed platelets. Although a traceable amount of FXIII-A could be detected in neutrophils of healthy individuals, the administration of NC9, a specific FXIII-A inhibitor^33^ did not affect the BPNH_2_ incorporation into cellular and NET proteins (data not shown). In line with this, isolated and PMA-activated neutrophils of a FXIII-A-deficient patient were able to form a functional NET structure and the level of BPNH_2_ incorporation was comparable to controls (Supplementary Figure 2B) suggesting that FXIII-A in human neutrophils does not contribute to NET formation.

### Mono- and polyamine treatment disrupt NET structure

**Figure 6:**
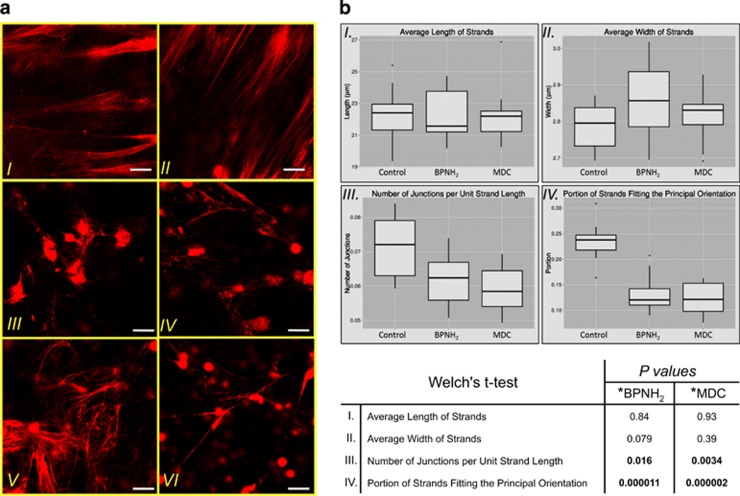
Monoamines and exogenously administered SPM disrupt normal NET structure. (**a**) Representative images of NET structures generated with PMA alone (I), or in the presence of MPNH_2_ (II), PNH_2_ (III), MDC (IV), BPNH_2_ (V) or SPM (VI) for 4 h. DNA was stained with SytoxOrange (red). The experiment was repeated at least four times with neutrophils from independent healthy donors, with similar results. The original magnification was × 60, the scale bar represents 10 *μ*m. (**b**) Quantitative evaluation of different parameters of the NET. The average length (I) width (II) of the NET strands, number of junctions per unit strand length (III) and the orientation of the DNA filament (IV) were quantified from control, BPNH_2_ and MDC treated NETs derived from four independent donors. At least 10 independent fields of view from each sample (donor and treatment) were used for the calculation. Data are presented with box and whisker plots and Welch's *t*-test was used for testing the equality of the means of any two samples. *P*-values are summarized in chart

In order to clarify the role of polyamine incorporation and protein cross-linking in NET formation and stabilization, we analyzed multiple parameters of normal and competitive inhibitor treated NETs. NET structure was dissected by Z-stacking in 0.5 *μ*m slices to a 50 *μ*m range above the layer of the attached cells. Monoamines with a reactive terminal amino group (PNH_2_, BPNH_2_ and MDC) significantly altered NET structure (Figure 6a). Differences especially concerned the organized orientation of NET filaments and density of the DNA strands, in addition to complete lack or diminished frequency of the thin DNA filaments (<0.5 *μ*m in width) upon monoamine treatment. SPM excess also disrupted NET structure and resulted in fewer strands of a thicker composition (Figure 6a, panel VI). MPNH_2_ had no significant effect on the structure (Figure 6a, panel II).

Computational analysis of the confocal microscopic images obtained by Z-stacking allowed us to objectively determine characteristic parameters of the NET structure. We identified a significant decrease in the average number of junctions per unit strand length upon monoamine and SPM treatment, whereas there was no detectable difference in the average lengths of the strands. In the average strand width, we observed increased values upon such treatments, although none of these were significant according to Welch's *t*-test (Figure 6b, panels I–II). Regarding the strand orientation, a significantly larger portion was directed in an unordered manner suggesting disorganization of the whole structure in response to the monoamine or SPM treatment (Figure 6b, panels III–IV).

### Disruption of NET structure compromises bacterial trapping

**Figure 7:**
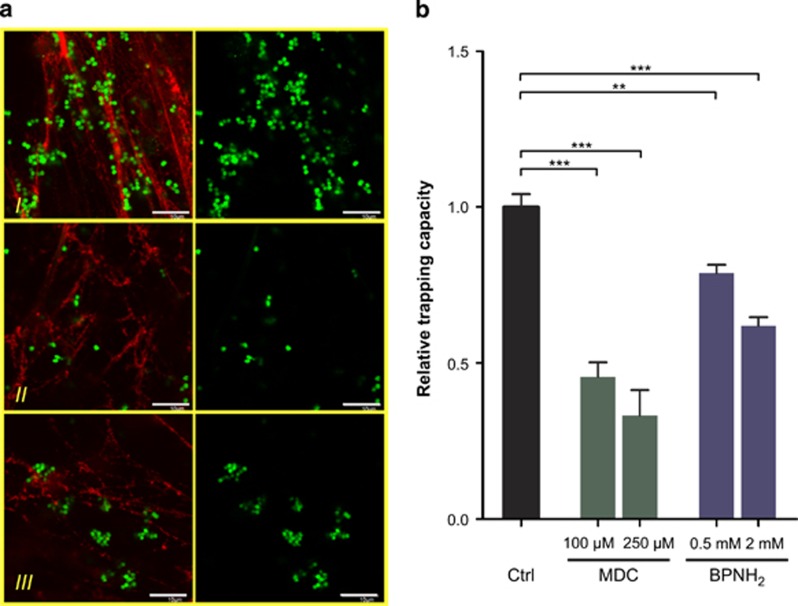
Disruption of NET structure by interfering endogenous polyamine incorporation by monoamines compromises bacterial trapping capacity of the NET. (**a**) Representative images of trapped *S. aureus* in NETs generated with PMA (I) or in the presence of MDC (II) or BPNH_2_ (III). Left panels show NET (red) and FITC-labeled trapped *S. aureus* (green); right panel shows *S. aureus* alone. Experiment was repeated three times with neutrophils from independent healthy donors, with similar results. Original magnifications were × 40 and scale bars represent 10 *μ*m. (**b**) Quantification of the trapping capacity of the NET by bacterial trapping assay. Neutrophils were plated and activated in an ELISA plate for 3 h with PMA (control), PMA and MDC (100 and 250 *μ*M) or PMA and BPNH_2_ (0.5 and 2 mM). Phagocytosis was blocked by cytochalasin D then FITC-labeled *S. aureus* was given to the cells in 1 : 100 neutrophil:bacterium ratio for additional 1 h. Unbound bacteria were washed away and fluorescence (proportional to the amount of the trapped bacteria) was determined by an ELISA reader. Experiment was repeated three times with neutrophils from independent healthy donors (*n*=3) with six technical parallels. Data represent mean±S.D. ***P<0.001, **P<0.01 as compared with controls by Student's *t-*test

To investigate functional consequences of disrupting the normal NET structure, we first visualized immobilized FITC-labeled *S. aureus* associated with NET. The organized NET structure of control neutrophils was able to capture large number of bacteria, whereas the amount of trapped bacteria was significantly reduced if the neutrophils were treated either with BPNH_2_ or MDC (Figure 7a). The trapping capacity of NETs derived from differently treated neutrophils was determined by bacterial trapping assay. Consistently with microscopic observations, neutrophils forming NET in the presence of either BPNH_2_ or MDC were able to immobilize significantly lower amount of bacteria compared with control cells (Figure 7b). Thus, we concluded that endogenous polyamine incorporation and protein–protein cross-links associated with the normal NET formation is essential for neutrophils to trap bacteria efficiently.

## Discussion

The observation of the reproducibly generated characteristic NET architecture with restricted protein composition^34, 35^ fostered us to investigate possible biochemical events that regulate NET formation. Polyamines and nuclear aggregates of polyamines are present in NET and have already been proposed to have possible role in stabilizing NET structure by interacting with its DNA backbone.^36^ In this study, we have demonstrated that covalent cross-linking of the protein content of the NET is an integral and stabilizing constituent of functional NET formation. Owing to their multiple reactive amino groups, it seemed conceivable that polyamines could act as linker agents resulting in intermolecular protein–protein cross-link formation. Indeed, utilizing specific antibodies to polyamines we could demonstrate the presence of covalently linked polyamines in the protein content of the NET by immunohistochemistry and ELISA. Stimulation of neutrophil oxygen metabolism with PMA in the presence of primary amines, including polyamines, resulted in oxidative amine incorporation into proteins.^4, 37^ The process involves MPO-catalyzed oxidation of chloride to HOCl in the presence of hydrogen peroxide, followed by chlorination of exogenously added amines as well as endogenous cellular amines to yield nitrogen–chlorine derivatives, which can modify proteins in neutrophils.^4, 37^ Here, we have shown that chlorinated polyamines get covalently incorporated into NET proteins by a process that we found not affected by the inhibitory effect of mono- and polyamines on ROS generation.^26, 27^ Furthermore, polyamines chlorinated at both primary amino groups can form cross-links between NET proteins contributing to NET stability. This may also explain why MPO is required for proper NET formation.^7^ Based on our findings, we propose that during NET formation MPO, beyond facilitating chromatin de-condensation in conjunction to elastase in the nucleus,^38^ is preferentially located at DNA strands to form HOCl that subsequently generates chlorinated polyamines with cross-linker potential. Chlorinated polyamines then promote covalent linkage among specifically assembled proteins destined to be selectively released with DNA strands of the NET.

Polyamines are natural substrates of TGases and TGase-mediated incorporation of polyamines into proteins occurs in cells and tissues modifying, for example, cornification in the skin, lens crystallines, functions of actin, tubulin, RhoA, histones and heat shock proteins.^22, 29, 30, 39, 40, 41, 42^ Cornification and crystallin modifications are particularly relevant to this study as in both processes TGase-mediated formation of bis(*γ*-glutamyl)polyamine cross-links between proteins by TGase 1 and 2, respectively, was observed^29, 30, 40^ in parallel to protein cross-linking through *ɛ*(*γ*-glutamyl)lysine bonds. NET protein fractions contain both bis(*γ*-glutamyl)polyamine and *ɛ*(*γ*-glutamyl)lysine bonds suggesting that TGase-mediated cross-linking contributes to selective enrichment of specific proteins in the NET and its stabilization. In line with these data, we observed Ca^2+^-dependent TGase activity using PUT and BNPH_2_ during NETosis. Thus, besides Ca^2+^-independent, chlorination-mediated amine incorporation, TGase activity contributes to cross-link formation stabilizing NET structure.

We found that FXIII-A and TGase 1 are present in neutrophils. However, neutrophils isolated from a FXIII-A-deficient patient could incorporate primary amines into proteins during NETosis to a similar degree as controls making it unlikely that this FXIII-A has a major role in cross-link formation. TGase 1 has a pivotal role in the cornification process ^43^ cross-linking a set of proteins expressed during terminal differentiation of keratinocytes. According to our presented data, TG1 is also expressed in circulating neutrophils where it is cleaved and activated by a so far not identified protease. TG1 is one of the least restricted TGases in glutamine substrate specificity and we suggest it can serve, in addition to chlorinated polyamines, as a cross-linker enzyme assembling and stabilizing a broad set of NET proteins.

Surprisingly, the SDS-polyacrylamide electrophoretic separation pattern of the NET proteins prepared from stimulated neutrophils of different individuals was highly reproducible but the composition of the protein bands was heterogenic containing polypeptides with molecular weights different from what was expected suggesting their extensive posttranslational processing during NET formation involving both cross-link formation and proteolysis. The analysis of cross-links formed by chlorinated polyamines and TGase-dependent reactions between the proteins (Figure 4) indicates the formation of an extensive and specific cross-linking pattern keeping these proteins or their modified and processed forms together. The highly standard electrophoretic band pattern of the NET revealed by consecutive separations and the fact that the SPM and BNPH_2_ treatments did not alter it significantly but rather resulted in minor changes in protein composition indicates a robustness of the NET-forming biochemical systems. The nature of the obtained data and the cross-link analysis software cannot provide reliable quantitative information regarding the precise number of cross-links formed;^28^ in the experimental setup used only an approximation can be done. The variety of the position of the amino-acid residues participating in cross-link formation can be the result of extensive three-dimensional structure modifications caused by protease action (especially NE and cathepsin G) and citrullination.^44^ Converting the positively charged arginine to uncharged citrulline can destroy the functional structure of the proteins or can cause conformational changes leading to surface accessibility of otherwise buried amino acids permitting cleavage or cross-link formation.^45^ Limited proteolysis in a very similar manner as citrullination can lead to conformational changes^46^ making residues available for citrullination or cross-link formation. It is not known which of these processes happen first and in which extent they participate in NET formation. Much more accurate mass spectrometry analyzes are needed to get in-depth qualitative and quantitative information on the effect of citrullination, proteolysis and cross-link formation on the NET structure.

To investigate NET functionality when polyamine and TGase-dependent cross-linking is prevented, we used aliphatic monoamines, including BPNH_2_, which can interfere with both non-enzymatic and TGase-dependent cross-link formation. Monoamines get incorporated into cell and NET-specific proteins during NETosis as demonstrated *in situ* with immunohistochemistry, in cell lysate and isolated NET protein preparations. We observed a different labeling pattern when we compared cellular proteins with NET-specific proteins (Figure 3) indicating that protein mono- and polyamination occur at a biochemically regulated manner selectively targeting a fraction of the protein in NETosing neutrophils. We presumed that endogenous polyamines and monoamines may compete for specific amine binding sites on the surface of proteins. Indeed, parallel with the exogenous monoamine incorporation, we observed significant reduction in the level of the endogenous polyamine incorporation. Hence, monoamines are potent inhibitors to reduce the level of endogenous polyamine incorporation as well as to modify the polyamine and *ɛ*(*γ*-glutamyl)lysine cross-linking processes (Figure 4). Using monoamines as cross-linking inhibitors, we observed significantly altered NET structure. Furthermore, overloading NETosing neutrophils with exogenous SPM also lead to disturbed NET architecture. Computational analysis revealed that changes in NET structure particularly affected width and the orientation of the NET filaments and the frequencies of the junctions per unit strand (Figure 6). The covalently incorporated polyamines into the protein globules of the NET may also serve with their internal positive charge as anchor points for the DNA strands and disturbing the regulated pattern of polyamine linkers could distort the orientation and the charge-driven bundling of DNA in the NET. Importantly, inhibition of endogenous polyamine incorporation and interfering with the cross-linking process lead to impaired pathogen trapping capacity of the NET through the disturbance of its natural structure. These results indicate that endogenously adjusted covalent polyamine conjugation and cross-linking of NET-specific proteins contribute to the overall stability of NET and are essential for its biological function.

Extracellular histones have cytotoxic effect on endothelial cells and can cause fatal organ dysfunctions through microvascular necrosis.^47, 48, 49^ They act as damage-associated molecular patterns and trigger innate immune response either via the NOD-like receptor family, pyrin domain containing 3 (NLRP3) inflammasome or various TLR pathway activations that also could lead to tissue injury.^48, 50, 51^ Furthermore, extracellular histones induce platelet aggregation and thrombus formation.^52, 53, 54^ Recently, neutrophils and NET formation have been also associated with autoimmune manifestations: PAD4 catalyzed histone citrullination increases their antigenicity and potentially leads to autoimmunity (e.g., rheumatoid arthritis, Felty's syndrome).^55, 56^ Normally, DNA content of the induced NET is rapidly degraded by DNAases and scavenged from the circulation by professional cells. Compromised clearance and persistent exposure of DNA together with the associated histones in susceptible individuals enforces autoantigen presentation and leads to expansion of autoreactive lymphocyte clones and consequent autoantibody production as exemplified in lupus.^57, 58, 59^ Based on our findings, we also propose that in analogy to protein cross-link formation in apoptosis^60, 61^ NET-specific cross-links could prevent spreading of neutrophil intracellular contents and limit harmful release of proteins (histones, NE, MPO) as well as genomic DNA into the circulation. Localizing cytotoxic and immunostimulatory components of the NET to the site of the infection by regulated protein cross-linking – with the help of MPO-dependent formation of chlorinated polyamines and TGase activation – may significantly contribute to prevention of harmful consequences of the host response to invading microbes executed by NETosis.

## Materials and Methods

### Isolation of human peripheral neutrophils

For these studies, approval was obtained from the ethics committee of the Medical and Health Science Center, University of Debrecen, Debrecen, Hungary (DEOEC RKEB/IKEB Prot. No. 2745-2008). From healthy individuals, 50 ml of venous blood was collected into vacutainer tubes containing EDTA (BD Biosciences, San Jose, CA USA, 367525) and centrifuged 15 min, 2500 r.p.m at RT to remove plasma containing platelets. Cells (∼25 ml) were transferred into a 50 ml tube and 12 ml dextrane (Pharmacosmos A/S, Holbaek, Denmark, Dextran T500) (3% dextrane in saline solution) was given to the cells. The tube was filled up to 50 ml with saline solution and incubated for 30 min at RT. Supernatant containing white blood cells was collected and diluted 4x with phosphate-buffered saline (PBS)-EDTA (20 mM), then cells were centrifuged (1800 r.p.m., 7 min, 4 °C). The pellet was suspended in 3 ml PBS-EDTA and the cells were layered on a Histopaque (Sigma-Aldrich, St. Louis, MO, USA, 11191 and H8889) gradient (6 ml Hp 1119, 5 ml Hp 1077 in a 15 ml tube) and then centrifuged for 30 min, 1800 r.p.m. at 4 °C. The supernatant (containing lymphocytes and monocytes) was discarded; neutrophils were collected and washed 2x with 50 ml PBS-EDTA (20 mM) to remove the rest of the platelets.

### Induction of NET formation and amine treatment

Isolated human neutrophils were seeded in 6-, 12-, 24- or 96-well tissue culture plate to a density of 2.0 × 10^6^/ml medium (RPMI 1640) (Sigma, R5886) and activated with 20 nM PMA (Sigma, P8139) or with *S. aureus* (1 : 10 neutrophil:bacterium ratio) for 4 h at 37 °C in a 5% CO_2_ atmosphere. Mono- and polyamine treatment were carried out as follows: SPM (Sigma, S3256) 2 mM; methylamine (Sigma, 426466) 2 mM; PNH_2_ (Sigma, 171409) 2 mM; MPNH_2_ (Sigma, 496138) 2 mM, BPNH_2_ (Zedira, B002) 2 mM for 4 h.

### Detection of NET with laser scanning confocal microscopy

Neutrophils were seeded in eight-well coverslip chambers at 5 × 10^5^ cell/200 *μ*l per well density with or without induction of NET formation induced by PMA (20 nM, 4 h). After 4 h, cells were fixed with 4% paraformaldehyde (Sigma, P6148) in PBS (10 min, RT) and DNA content of the cells and NET was stained either with SYTO 83 Orange or SYTO green fluorescent nucleic acid stain (Invitrogen/Thermo Fisher Scientific, Waltham, MA, USA, S11364, S34854).

### Purification of NET proteins

Human neutrophils were seeded in six-well tissue culture plates to a density of 2.0 × 10^6^/ml in 4 ml medium/well (RPMI 1640) and activated with 20 nM PMA for 4 h at 37 °C in a 5% CO_2_ atmosphere. Each well was carefully washed after removing the supernatant by pipetting 1 ml of pre-warmed protease inhibitor cocktail (Sigma-Aldrich, P8340) containing RPMI into the well along the wall of the well and incubated for 5 min at 37 °C. Subsequently, the NETs were digested for 20 min in 1 ml RPMI with 10 U/ml DNase-1 (Worthington, Biochemical Corp., Lakewood, NJ, USA, LS006333). Supernatants were collected on ice and the samples were centrifuged at 16 000 × *g* at 4 °C to remove debris. In all, 0.5 ml of supernatant was mixed with 1.5 ml of ice-cold acetone (−20 °C) in a 2 ml Eppendorf tube and incubated overnight at −20 °C to precipitate protein content. Samples were centrifuged at 10 000 × *g* for 30 min at 4 °C then matched protein pellets were solubilized in 200 *μ*l Laemmli buffer+50 *μ*l 5x-SDS loading buffer, denatured at 100 °C for 10 min and used for further analysis.

### Quantification of polyamines incorporated into proteins with ELISA

In all, 100 *μ*l of neutrophil cell lysate (1–5 *μ*g/*μ*l protein in Laemmli buffer) was incubated in 96-well ELISA plate (maxisorb Nunc plate) for 2 h at RT to bind neutrophil proteins onto the plate surface. Wells were washed with 200 *μ*l PBS/Tween (0.1%) and blocked by incubating with 200 *μ*l PBS/Tween (0.1%) supplemented with 5% bovine serum albumin (BSA) and 5% goat serum (Sigma-Aldrich, G9023) for 1 h at RT. After washing the wells with 200 *μ*l PBS/Tween (0.1%), 100 *μ*l of rabbit anti-SPM antibody (Covalab, S.A.S., Villeurbanne, France, pab0022) or rabbit serum (Covalab) as negative control was given to the wells in 1 : 100 dilution in PBS/Tween (0.1%) supplemented with 5% BSA and 5% goat serum for 2 h at RT. Wells were washed 3x with 200 *μ*l PBS/Tween (0.1%) and followed by secondary antibody (horseradish peroxidase (HRP)-conjugated anti-rabbit antibody) (Covalab, lab0273) in 1 : 1000 dilution in 100 *μ*l PBS/Tween (0.1%) for 1 h at RT. Wells were washed 3x with 200 *μ*l PBS/Tween (0.1%) and incorporated polyamines were detected using 100 *μ*l substrate solution (3,3',5,5'-tetramethylbenzidine ready-to-use substrate solution, Sigma, T4444) for 5–10 min at RT. Reaction was determined by adding 50 *μ*l 2 N sulfuric acid and optical density was measured at 450 nm by an ELISA reader.

### Protein purification and western blot

Resting and NETosing neutrophils were collected, washed with cold PBS-EDTA (20 mM) and lysed in cell lysis buffer (10 mM Tris, pH 7.4, 100 mM NaCl, 1 mM EDTA, 1% Triton X-100, 10% glycerol, 0.1% SDS). Cell lysates were denaturated in a buffer containing 0.125 m Tris-HCl, pH 6.8, containing 4% SDS, 20% glycerol, 10% mercaptoethanol and 0.02% bromophenol blue at 100 °C for 10 min. In all, 20 *μ*g protein from each sample were electrophoresed in 10% SDS-polyacrylamide gels and electroblotted onto PVDF membranes. The blots were first saturated with 5% BSA in Tween 20-tris-buffered saline (TTBS). Then, mouse monoclonal antibody against either TG2 (Thermo Fisher Scientific, Waltham, MA, USA, MS-224-P) or rabbit polyclonal antibody against TG1 (Zedira, Darmstadt, Germany, A018), diluted in 0.5% BSA in TTBS 1 : 5000 or 1 : 1000, respectively, was added and incubated at 4 °C overnight, followed by incubation with HRP-labeled affinity-purified goat anti-mouse IgG (Covalab, lab0252) or anti-rabbit IgG (Covalab) in TTBS 1 : 5000 at room temperature for 1 h. Each step was followed by 3 × 15-min washes in TTBS. FXIII-A was visualized by ECL Kit (Advansta, Menlo Park, CA, USA, K-12045-D50).

### Immunofluorescent staining

Neutrophils were seeded in eight-well coverslip chambers at 5 × 10^5^ cell/200 *μ*l per well density with or without induction of NET formation induced by PMA (20 nM, 4 h) or *S. aureus* (1 : 10 ratio, 4 h). After 4 h, cells were fixed with 4% paraformaldehyde (Sigma, P6148) in PBS (10 min, RT), and then permeabilized with 0.1% Triton X-100 (Sigma, X100) in PBS (20 min) and blocked with TTBS, 5% BSA for 20 min. Cells were incubated with TTBS 1% BSA containing either mouse monoclonal antibody against TG2 (Thermo Scientific) or rabbit polyclonal antibody against TG1 (Zedira) and FXIII-A (Zedira, A016) for 2 h RT; washed three times for 5 min in TTBS-Tween-20 (0.1%). Alexa Fluor-647-conjugated goat anti-mouse or anti-rabbit secondary antibody (Life Technologies, A-21235, A-20991) were used for 1 h at room temperature to visualize TG1, TG2 and FXIII-A. To detect protein-bound SPD and SPM in cells and NET structure, rabbit anti-SPM antibody (Covalab, pab0022) or rabbit serum (Covalab) as negative control was used in 1 : 50 dilution in TTBS 5% BSA and 5% goat serum (Sigma-Aldrich) for 8 h at 4 °C followed by the incubation of Alexa Fluor-647-conjugated goat anti-rabbit secondary antibody (Life Technologies, A-20991) for 1 h at RT. Protein-bound BPNH_2_ were detected using Alexa Fluor-647-conjugated streptavidin (Life Technologies, S32357). DNA content of the cells and NET was stained as described in Detection of NET with laser scanning confocal microscopy.

### Detection of monoamine incorporation to proteins by western blot

NET formation of seeded neutrophils was induced either in the presence of 2 mM BPNH_2_ (Zedira, B002) or 100 *μ*M MDC (Sigma, 30432). In order to test the role of calcium ion in the incorporation of the TGase substrate (BPNH_2_), NET formation was induced for 2 h followed by administration of EDTA (1 mM) for 30 min in order to chelate Ca^2+^ ions and finally BPNH_2_ was added for an additional 3 h. After incubation, cells were harvested, protein extracts were generated and electrophoresed as described in Protein purification and western blot section. Protein-bound BPNH_2_ was detected using HRP-labeled streptavidin (Pierce/Thermo Fisher Scientific, 21130) after electroblotting.

### Measurement of protein concentrations

Total protein concentration of NET samples was determined with Bradford (Sigma) method.^62^ The BSA (Sigma) was used as a standard in the protein assay.

### SDS-polyacrilamide gel electrophoresis

In all, 20 *μ*g of NET protein was separated on 12% polyacrylamide gel in a Mini Protean Tetra Cell (Bio-Rad, Hercules, CA, USA). Electrophoresis was performed for 1.5 h at 30 mA constant current and ProSieve Protein Ladder (Lonza, Basel, Switzerland) was used as a protein molecular weight marker. Protein staining was performed using PageBlue Protein Staining Solution (Thermo Scientific), gels were scanned by Pharos FX Plus Molecular Imager (Bio-Rad) and the image analysis was done using QuantityOne Software (Bio-Rad).

### In-gel digestion

Protein bands excised from gels were cut into smaller pieces and dried in speed-vac. Gel pieces were destained using 25 mM ammonium bicarbonate pH 8.5:50% acetonitrile (ACN) (1 : 1) solution, followed by reduction with 20 mM dithiothreitol (Bio-Rad) for 1 h at 56 °C. The alkylation was done using 55 mM iodoacetamide (IAA) (Bio-Rad) for 45 min at room temperature in the dark and the digestion was carried out overnight at 37 °C using 100 ng stabilized MS grade trypsin (ABSciex, LLC, Framingham, MA, USA). The reaction was stopped by adding formic acid (FA) (VWR, Radnor, PA, USA) and the tryptic peptides were extracted from the gel pieces, concentrated and re-dissolved in 10 *μ*l 1% FA and used for mass spectrometry-based protein identification.

### Protein identification by mass spectrometry

The digested samples were analyzed by reversed-phase liquid chromatography coupled to nanospray tandem mass spectrometry (LC-MS/MS) using a 4000 QTRAP (ABSciex) mass spectrometer operated by Analyst software 1.4.2 (ABSciex). The chromatographic separation was performed on a Proxeon EASY-nLC II (Bruker, Billerica, MA, USA) at a flow rate of 300 nl/min during a 90-min gradient. The mobile phase A was 0.1% FA in LC-MS grade water (Sigma), whereas the mobile phase B was ACN with 0.1% FA. The peptide mixture was first loaded to a 5 × 0.3 mm desalting column packed with 5 *μ*m C18 resin (Zorbax 300SB, Agilent, Santa Clara, CA, USA), followed by separation on a 150 mm × 75 *μ*m analytical column (3.5 *μ*m pore size C18 resin, Zorbax 300SB, Agilent). The nano-flow system was connected to NanoSpray II MicroIon source, the spray voltage was 2800 V, ion source gas was 50 psi, the curtain gas was 20 psi and the source temperature was 70 °C.

The 4000 QTRAP mass spectrometer was operated in positive mode and information-dependent acquisition was administrated. After the first mass scan (300–1700 amu), the charge of the precursor ion was established using Enhanced Resolution (ER) scan, then the MS/MS spectra of the two most intensive ions (mass range 100–1900 amu) were recorded. Two MS/MS spectra were summed in each case and used for protein identification. For protein identification, ProteinPilot 4.5 software (ABSciex) was administrated using the Paragon algorithm and the UniProtKB/Swiss-Prot database (2014. 06. 11. version, 545 536 entry). Peptide sequences having at least 95% confidence were accepted and for protein identification at least two peptides were used.

### MS/MS-based investigation of cross-linked NET proteins by StavroX software

StavroX 3.2.10 software was used to identify the possible cross-links among the NET proteins. For the analyzes, MS/MS data were loaded as Mascot generic format (mgf) files including each recorded spectrum. Amino-acid sequences of previously identified NET proteins were imported from UniProtKB in FASTA format. Enzymatic cleavage sites of trypsin were determined as C-terminal to lysine (Lys) and arginine (Arg) and blocked by proline (Pro). Variable modifications were defined such as oxidation of methionine (Met) and carbamidomethylation of cysteine (Cys). SPM, SPD and PUT were used as probable crosslinkers, searching for the presence of the following cross-links: (1) SPM, SPD and PUT chloramines are formed upon MPO activity. The chloramines react with protein-bound lysines forming Lys-polyamine-Lys cross-links (mass change: 200.2, 143.14 and 86.08 Da, respectively). (2) The chloramines react with protein-bound lysines or methionines forming Lys-polyamine-Met cross-links (mass change: 200.2, 143.14 and 86.08 Da, respectively). (3) SPM, SPD and PUT form cross-links between protein-bound glutamines (Gln-polyamine-Gln) in a reaction catalyzed by the TGase (mass change: 166.19, 109.14 and 54.09 Da, respectively). TGase catalyzes the formation of isopeptide bonds between protein-bound lysines and glutamines, so the presence of Gln-Lys cross-links (mass change: 17.03 Da) was also checked. The calculated mass changes were implemented into the software and the MS/MS spectra were searched for b- and y- ions corresponding to each possible cross-links. The results were manually investigated and those hits were accepted where the score value of the identified cross-linked proteins was positive and the b- or y- ion series contained at least four ions. The sequence of the cross-linked peptides and the position of the cross-link was exported to Excel files and used for the visualization of the network of cross-linked NET proteins.

### TG1-specific activity assay

Specific TG1 enzyme activity was evaluated in neutrophil and HaCaT cell lysate (provided by Dr. Máté Demény, University of Debrecen) alone or in the presence of 1 mM nonspecific irreversible TGase inhibitor IAA^63^ (Sigma, I6125) or 10 *μ*M specific irreversible TG2 and FXIII-A inhibitor (ZDON) (Zedira, Z006) using the TG1-CovTest (Covalab), which utilize immobilized SPM as second substrate of TG and the biotin-YEQHKLPSSWPF peptide (pepK5) as preferred first substrate for TG1, respectively. The assay was performed following the manufacturer's instructions. In brief, 50 *μ*l of the reaction buffer was mixed with 50 *μ*l samples and incubated in the microtiter plates at 37 °C for 30 min, and at the end of this period, wells were emptied and washed three times with 0.1m Tris-HCl, pH 8.5 containing 0.1% Tween 20. The incorporated biotinylated peptides were detected using streptavidin peroxidase (R&D Systems, Inc., Minneapolis, MN, USA) and the peroxidase substrate 3,3′,5,5′-tetramethylbenzidine (Sigma). The reaction was stopped by the addition of 2 N H_2_SO_4_ and the absorbance read on a microplate reader at 450 nm.

### Bacterial trapping assay

Neutrophils were seeded in 96-well culture plate (0.5 × 10^6^ cells/200 *μ*l per well) in pure RPMI medium and treated with cytochalasin D (10 *μ*g/ml) (Sigma, C8273) to prevent phagocytosis, PMA (20 nM) and MDC (100, 250 *μ*M) or BPNH_2_ (0.5, 2 mM) or left untreated. Plate was centrifuged at 800 *g* for 10 min and incubated for 3 h at 37 °C to induce NET formation. FITC-labeled *S. aureus* was given to cells in 10 *μ*l RPMI in 1 : 100 cell:bacterium ratio (0.5 × 10^8^ bacterium per well) and incubated for 1 h at 37 °C. Plate was gently washed three times with 200 *μ*l cold PBS to remove untrapped bacteria and fluorescence was measured by a fluorescence plate reader (excitation 485 nm, emission 528 nm). For confocal microscopic analysis, neutrophils were seeded in eight-well coverslip chambers and treated similarly. After washing untrapped bacteria away, cells were fixed with 4% paraformaldehyde and DNA was stained with SYTO 83 orange fluorescent nucleic acid stain (Invitrogen, S11364).

### Detection of ROS

NADPH oxidase-derived superoxide (O2−) and other ROS productions of neutrophils were determined by a L-012-based chemiluminescence assay using BioT Synergy H1 microplate reader (Winooski, VT, USA). Measurements were performed in Cornin Costar 96-Well White Solid Plates in which the reaction volume of 100 *μ*l contained 5 × 10^4^ cells and 100 *μ*M L-012. Neutrophils were stimulated with PMA (50 nM) in the presence of different mono- or polyamines (methylamine, MNH_2_; PNH_2_; MPNH_2_ and SPM) and the chemiluminescence was monitored from 2.5 min for 90 min.

## Abbreviations

ABAH: aminobenzoic acid hydrazide
AZU1: azurocidin
BPNH_2_: 5-(biotinamido)-pentylamine
EDTA: ethylenediaminetetraacetic acid
HOCl: hypochlorous acid
HRP: horseradish peroxidase
IAA: iodoacetamide
LC-MS: liquid chromatography–mass spectrometry
MDC: monodansylcadaverine
MPNH_2_: *N*-methylpentylamine
MPO: myeloperoxidase
NE: neutrophil elastase
NET: neutrophil extracellular trap
NLRP3: NOD-like receptor family, pyrin domain containing 3
PAD4: peptidylarginine deiminase 4
PBS: phosphate-buffered saline
PMA: phorbol-12-myristate 13-acetate
PNH_2_: pentylamine
PUT: putrescine
ROS: reactive oxygen species
SDS: sodium dodecyl sulfate
SPD: spermidine
SPM: spermine
TGase: transglutaminase
TLR: Toll-like receptor
TTBS: Tween 20-tris-buffered saline
