# Supplementary figures and images for: Protein cross-linking by chlorinated polyamines and transglutamylation stabilizes neutrophil extracellular traps
Source: Cell Death Dis. 2016 Aug 11;7(8):e2332–. doi: 10.1038/cddis.2016.200 (PMC5108309; doi:10.1038/cddis.2016.200)

Supplementary Figure 1

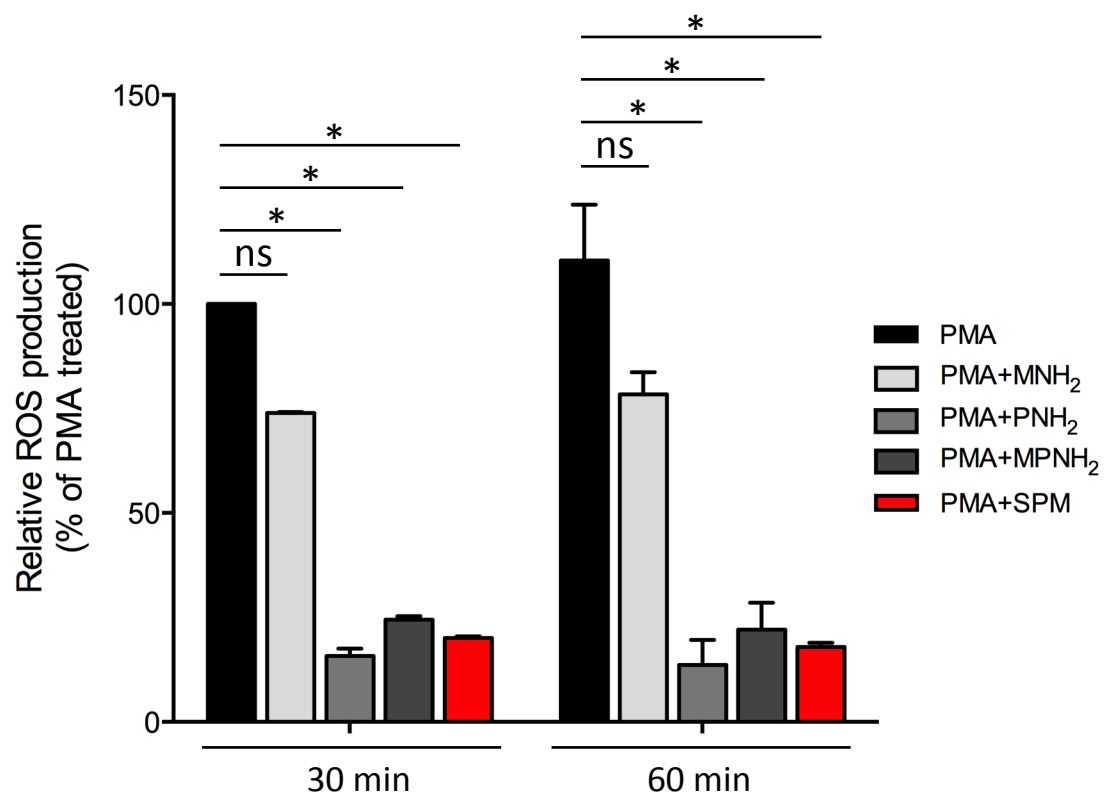

Supplement: Supplementary Figure 1 [file cddis2016200x1.pdf]

## Supplementary Figure 2

A

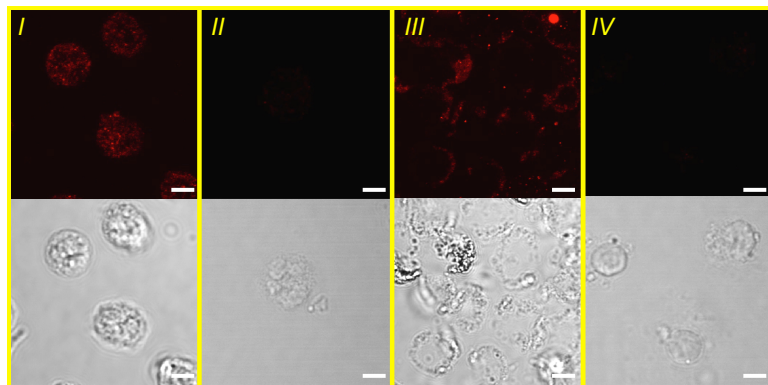

B

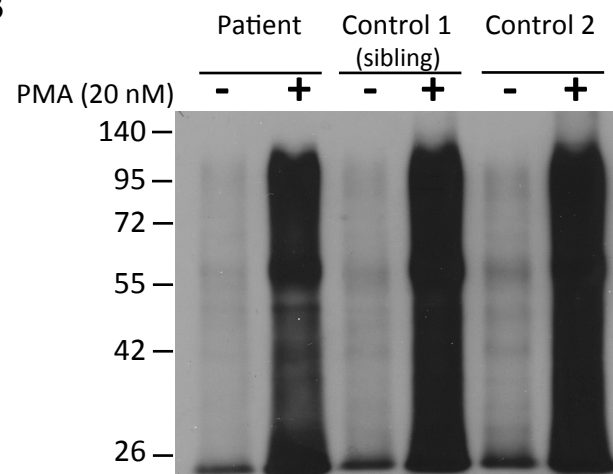

Supplement: Supplementary Figure 2 [file cddis2016200x2.pdf]
