# Supplementary material for: Protein cross-linking by chlorinated polyamines and transglutamylation stabilizes neutrophil extracellular traps
Source: Cell Death Dis. 2016 Aug 11;7(8):e2332–. doi: 10.1038/cddis.2016.200 (PMC5108309; doi:10.1038/cddis.2016.200)
Supplement: Supplementary Figure and Table Legends [file cddis2016200x5.docx]

**Supplementary Table 1. List of proteins identified in each band.** The name and accession numbers of the identified proteins and the sequence coverage (%Cov) are listed. The identified peptide sequences for each protein are indicated along with the confidence values of sequence identification (Conf), the post-translational modifications (Modifications) (@ indicates the site of modification), the theoretical molecular weights and m/z values, the recorded precursor mass and m/z values and the delta mass (dMass) values. Data available at <http://ngsdebftp.med.unideb.hu/proteomics/>.

**Supplementary Table 2. List of crosslinks between the identified proteins.** The type and position of crosslinks are listed for identified proteins. The position and the sequence surroundings of amino acids participating in the crosslinks are indicated. In case of each protein the gene symbols are used. * indicates that based on the available data either of the amino acids can participate in crosslink formation. Data available at <http://ngsdebftp.med.unideb.hu/proteomics/>.

**Supplementary figure 1. Effect of mono- and polyamines on PMA-induced ROS production by human neutrophils**

NADPH oxidase-derived superoxide (O2·−) and other reactive oxygen species (ROS) productions of neutrophils were determined by a L-012-based chemiluminescence assay using BioT Synergy H1 microplate reader. Measurements were performed in Cornin Costar 96-Well White Solid Plates in which the reaction volume of 100 μL contained 5 × 104 cells and 100 μM L-012. Neutrophils were stimulated with PMA (50 nM) in the presence of different mono- or polyamines (methylamine, MNH2; pentylamine, PNH2; methyl-pentylamine, MPNH2 and spermine, SPM) and the chemiluminescence was monitored from 2.5 min for 90 min. Figure summarizes ROS production after 30 and 60 min. Data are means ± SEM (from 2 independent donor, measured with 5 technical parallels).

**Supplementary figure 2. Detection of FXIII-A in neutrophils and the effect of the FXIII-A specific inhibitor (NC9) on BNPH_2_ incorporation during NETosis. A.** Representative images of immunohistochemistry analysis of FXIII-A in human neutrophils. Resting neutrophils from a healthy individual (I) and a FXIII-A deficient patient (II) and PMA-activated neutrophils from healthy (III) and FXIII-A deficient subjects (IV) were stained with anti-FXIII-A rabbit polyclonal antibody and Alexa Fluor 647-conjugated goat anti-rabbit secondary antibody (red). The experiment was repeated four times with neutrophils from independent healthy donors and from one FXIII-A deficient patient. Lower panels show bright field images; the original magnification was 60×, the scale bar represents 5 µm. **B.** Detection of BPNH_2_ incorporation into cellular proteins in resting and activated neutrophils by Western blot. Neutrophils from a FXIII-A deficient patient and two healthy donors (control 1 – healthy sibling of the patient; control 2 – independent healthy donor) were preincubated with BPNH_2_ and then stimulated with PMA or left unstimulated for 4 hours (unstimulated controls). The levels of BPNH_2_ incorporation into cellular proteins were determined from cell lysates by Western blot.
